# Role of Glial Cells in Neuronal Function, Mood Disorders, and Drug Addiction

**DOI:** 10.3390/brainsci14060558

**Published:** 2024-05-30

**Authors:** Yousef Tizabi, Bruk Getachew, Sheketha R. Hauser, Vassiliy Tsytsarev, Alex C. Manhães, Victor Diogenes Amaral da Silva

**Affiliations:** 1 Department of Pharmacology, Howard University College of Medicine, 520 W Street NW, Washington, DC 20059, USA; bruk.getachew@howard.edu; 2 Department of Psychiatry, Indiana University School of Medicine, Indianapolis, IN 46202, USA; 3 Department of Neurobiology, University of Maryland School of Medicine, Baltimore, MD 21201, USA; tsytsarev@umaryland.edu; 4 Laboratório de Neurofisiologia, Departamento de Ciências Fisiológicas, IBRAG, Universidade do Estado do Rio de Janeiro, Rio de Janeiro 20550-170, RJ, Brazil; 5 Laboratory of Neurochemistry and Cell Biology, Department of Biochemistry and Biophysics, Institute of Health Sciences, Federal University of Bahia, Salvador 40110-100, BA, Brazil; vdsilva@ufba.br

**Keywords:** astrocytes, glial cells, gut microbiota, major depressive disorder, microglia, neuroinflammation, neurotrophic factors, oligodendrocytes, substance use disorder, synantocytes

## Abstract

Mood disorders and substance use disorder (SUD) are of immense medical and social concern. Although significant progress on neuronal involvement in mood and reward circuitries has been achieved, it is only relatively recently that the role of glia in these disorders has attracted attention. Detailed understanding of the glial functions in these devastating diseases could offer novel interventions. Here, following a brief review of circuitries involved in mood regulation and reward perception, the specific contributions of neurotrophic factors, neuroinflammation, and gut microbiota to these diseases are highlighted. In this context, the role of specific glial cells (e.g., microglia, astroglia, oligodendrocytes, and synantocytes) on phenotypic manifestation of mood disorders or SUD are emphasized. In addition, use of this knowledge in the potential development of novel therapeutics is touched upon.

## 1. Introduction

Mood disorders, as well as drug addiction, are major neuropsychiatric conditions with staggering medical, social, and financial costs. Mood disorders account for one of the highest work force disabilities worldwide [1]. Similarly, the burden attributed to alcohol and illicit drug use, excluding smoking, is estimated to result in the loss of nearly 5 percent of total life span [2]. Moreover, substance use disorder (SUD) is co-morbid with other psychiatric disorders such as anxiety, major depressive disorder (MDD), bipolar disorder, conduct disorder, and schizophrenia, and can result in unemployment, domestic violence, loss of productivity, and even suicide [3,4,5,6].

Although modest treatments for both disorders are available, incidences of treatment-resistant depression and relapse to drug abuse are major medical challenges. Thus, more effective interventions are urgently needed. However, without a thorough understanding of the neurobiological substrates of these disorders, novel therapies would be hard to come by. Tremendous effort is being expended in this regard. Indeed, discoveries in the mid-1950s heralded a new era of intervention in depression based on the monoamine hypothesis of mood disorders. This hypothesis posited that decreases in the levels of monoamines such as dopamine (DA) and norepinephrine (NE) in the brain were the cause of depression [7,8,9]. Hence, several monoamine-based drugs such as iproniazid, a monoamine-oxidase inhibitor (MAOI) that had been used in the treatment of tuberculosis, and imipramine, the first drug in the tricyclic antidepressant family, offered substantial help to many patients suffering from depression. Later, in the 1980s, selective serotonin reuptake inhibitors (SSRIs) such as fluoxetine (Prozac) were introduced [10]. More recently (March 2019), ketamine, a glutamate N-methyl-D-aspartate (NMDA)-receptor antagonist, was approved for use in treatment-resistant depression [11,12]. It is now believed that in addition to NMDA antagonism, ketamine modulation of metabotropic mGluR5 receptors also contributes to its antidepressant effects [13]. Nonetheless, considering the relatively high rate of depressive mood relapse (more than 40%), novel interventions are urgently needed.

Similarly, limited options in combatting drug addiction in general, and opioid use in particular, evidenced by the recent surge in deaths due to opioid overdose, underscore the need for fuller understanding of the brain circuitry controlling this behavior. Recent advances in unraveling some of the complexities of the central nervous system (CNS) including elucidation of the role of glial cells in neuronal functions, especially in relation to neuroinflammation, have been encouraging. This is because neuroinflammation is now considered a main culprit in almost all neuropsychiatric/neurodegenerative disorders and targeting it may be of therapeutic value [14,15,16]. Thus, in this review, we provide an update on glial–neuron interactions vis-a-vis major depressive disorder and drug addiction. We specifically concentrate on the role of different glial cells in neuroinflammation and down-stream consequences relevant to depression and drug addiction. Moreover, potential exploitation of this knowledge for novel interventions is touched upon.

## 2. Major Depressive Disorder (MDD)

As of 2017, major depressive disorder, a chronic, recurring, and debilitating mental illness, was ranked as the leading cause of disability around the world [17]. The advent of antidepressants in the mid-1950s, followed by more effective medications in the following decades, provided remarkable pharmaceutical interventions in this devastating illness. However, the lack of universal response, delay in onset of action, as well as the side effects associated with all available drugs necessitate the need for the development of more efficacious medications. The recent introduction of ketamine for treatment-resistant depression has been a step in the right direction. Nevertheless, it is imperative to enhance our understanding of the neuronal dynamics and circuitries involved in mood regulation to be able to provide better treatments. The complexity of the system regulating this behavior is further compounded by diversity and intensity of the symptoms and variations from patient to patient.

Whereas genetics may provide the foundational platform for any behavioral function, the manifestation of the phenotype may be equally dependent on the triggering of environmental factors which can exert a direct or indirect influence via epigenetic changes [18,19]. Fortunately, the availability of animal models of MDD with construct, predictive, and face validity allows for the in-depth investigation of not only the neurocircuitry but also the molecular intricacy. This, in addition to advances in neuroimaging, can facilitate translation of the preclinical findings to human studies. Below, we start with the discussion of several notable achievements in the identification of major players in MDD such as 1. neurotrophic factors, 2. neuroinflammation, and 3. gut microbiota.

### 2.1. Neurotrophic Factors and MDD

Contrary to the original conviction that after brain maturation, no more neurons are generated, it was discovered in the 1970s that neurogenesis does occur in select brain areas. Neurogenesis refers to synaptogenesis, the formation and pruning of spines and dendrites and strengthening of synaptic connections. However, for this process to be fully expressed, actions of neurotrophic factors (NTFs) are required [20,21]. One of the most important and extensively studied neurotrophins is brain-derived neurotrophic factor (BDNF), which is crucial in modulating neuronal differentiation, growth, survival, repairment, and plasticity [20,22,23,24]. Neuroplasticity is an umbrella term that refers to the ability of the brain to change and adapt following learning, experience, memory formation, and insults. This adaptation may include functional, structural, as well as molecular reorganization, which are essential not only for normal function, but also play a critical role in pathological conditions including neuropsychiatric diseases [25].

The crucial role of hippocampal BDNF in mood regulation has been amply documented [26,27,28,29,30]. Thus, it has been confirmed that dysregulation or impairment in BDNF could precipitate MDD [31,32]. Conversely, BDNF injection directly into the hippocampus imparts an antidepressant effect, and up-regulation of BDNF is often associated with an anti-depressant response [33]. More recently, it was proposed that circulating levels of BDNF may provide a biomarker for MDD [32].

It is of relevance to note that originally, only two major brain areas, the subventricular zone of the lateral wall of the lateral ventricle and the subgranular zone (SGZ) of the dentate gyrus (DG) of the hippocampus, with its projection to the olfactory bulb, were considered to contain neural stem cells (NSCs), and were referred to as “neurogenic niches” [34,35]. Lately, however, other, less-studied niches, such as the amygdala, the hypothalamus, and the cerebellum, have been added to the list of non-canonical areas [36].

The neurogenesis process is believed to occur in four stages. At stage 1, a pool of NSCs is formed which can subsequently differentiate into neuroblasts and immature neurons. At stage 2, the neuroblasts and immature neurons migrate to the granular cell layer. At stage 3, the immature neurons differentiate to mature neurons and establish synaptic connections with other neurons. At stage 4, the synaptic connections are established by mature neurons via their dendrites and axons, with other neurons and glial cells ensuring normal CNS functioning [37,38]. Indeed, newly born neurons in the DG of the adult brain contribute to the cognitive and emotional functions of the hippocampus [31,32,38].

Interestingly, a reciprocal relationship between BDNF and DA has been noted. Thus, early on, using genetically modified mice, it was shown that BDNF is responsible for maintaining the expression of DA D_3_ receptors [39,40]. This, in addition to the studies showing that BDNF enhances DAergic neurons’ survival, improving DAergic neurotransmission and motor performance, led to the suggestion that BDNF may play an important role in the pathophysiology of PD, mood disorders, and drug addiction [41,42,43]. More recently, it was reported that deep brain stimulation of the nucleus accumbens improves depressive-like behaviors via BDNF-mediated alterations of the DAergic pathway [44].

Conversely, it was demonstrated that in cultured embryonic mouse striatal cells, as well as in rat brain tissue, DA regulates BDNF expression [45,46]. BDNF action is mediated primarily via its high affinity TrkB (tyrosine kinase B) receptor, which leads to activation of the signal transduction cascades (IRS1/2, PI3K, Akt) [47,48]. A recent in vivo study demonstrated that DA modulates BDNF sensitivity and TrkB turnover in the medium spiny neurons of the striatum [49]. Curiously, a reciprocal interaction between BDNF and serotonin with implications in mood regulation has also been reported [50].

Taken together, it may be concluded that BDNF plays a central role in mood regulation via its interaction with the neurotransmitters implicated in this behavior.

### 2.2. Neuroinflammation and MDD

In addition to the roles of neurotransmitters and neurotrophic factors in MDD, the involvement of neuroinflammation is becoming a central theme in this disorder. Indeed, early on, an “inflammatory hypothesis” of depression was postulated based on the findings that elevated levels of circulating pro-inflammatory cytokines can lead to depressive symptoms [51,52,53]. Thus, it was observed that in individuals with depression, the concentrations of interleukin (IL)-6 and tumor necrosis factor (TNF)-α were higher than in non-depressed individuals. Conversely, plasma levels of IL-10, an anti-inflammatory cytokine, were negatively correlated with depressive symptoms [53,54]. Furthermore, it was proposed that mRNA of the pro-inflammatory cytokine IL-1-β may serve as markers for depression [55].

Persistent peripheral inflammation triggered by infection or immune disruption may lead to MDD. Thus, MDD is often co-morbid with systemic inflammatory diseases or conditions in which pro-inflammatory cytokines are overexpressed. These may include inflammatory bowel disease, allergies of different types, rheumatoid arthritis, cardiovascular disease, multiple sclerosis, diabetes, chronic liver disease, cancer, and even periodontitis, which is one of the most common chronic inflammatory disorders [56]. In these scenarios, during systemic inflammation, macrophages and monocytes are activated and release pro-inflammatory cytokines such as IL-1β, IL-6, and TNF-α, which in turn lead to microglial activation and an increase in central pro-inflammatory cytokines [56], which can precipitate MDD [33,56,57]. In addition, a high level of blood C-reactive protein (CRP), a specific inflammatory biomarker, was associated with greater MDD severity and a worse treatment response [58].

Interestingly, postpartum depression (PPD), a serious psychiatric disorder that dramatically affects women’s physical and mental health post-delivery, has been tied to inflammatory reactions [59]. PPD, in addition to affecting the emotion and cognition of the mother, can also damage the mother–-infant relationship, and delay the growth and mental health of the child [59,60]. Similarly, the association between stress and depressive mood may be mediated through inflammatory processes. Thus, it was revealed recently that following stress, the expression of claudin-5, a protein regulating the blood brain barrier (BBB), is reduced, leading to the penetration of IL-6 and induction of depression [61]. Chronic stress can also lead to increased inflammatory cytokines, which can also cause impairment in BDNF function and neurogenesis, leading to neurodegenerative and/or neuropsychiatric disorders including MDD [52,62]. More recent studies also attribute post-stroke depression to neuroinflammation induced by microglia and astrocytes responses [63,64].

Finally, regarding the fast-acting antidepressant, ketamine, it was shown that in addition to its role in glutamatergic modulation, as an N-methyl-D-aspartate (NMDA) receptor antagonist, it also promotes BDNF [65,66] sand and possesses anti-neuroinflammatory properties. The latter is manifested through its interaction with the complement system, which is an integral component of the innate immune system and has been implicated in the pathophysiology of depression [67].

### 2.3. Gut Microbiota and MDD

In the last few years, the gut microbiota (GM) and its connection to the brain, referred to as the gut–brain axis (GBA), has become an area of intensive research in the last few years. Although from the earliest centuries the gastrointestinal system was suspected as the root cause of physical and mental illnesses, it is only recently that GBA has begun to be touted as a potential therapeutic target [68]. This approach has been validated by the observation that the GBA closely interacts with the immune system and can exert profound influence on inflammatory processes. Thus, the role of the GBA in maintaining mental health in general, and MDD in particular, has been verified by numerous studies. It is of relevance to note that GM, in the human, harbors a range of 2000 bacterial species where its genomic content (microbiome) is slightly higher than the entire human genome [69,70,71].

Dysbiosis or perturbation of the GM can not only alter the neurotransmitters and neurotrophic factors involved in mood regulation but can also affect the immune system, leading to neuroinflammation, all of which can precipitate MDD [57,72,73,74,75]. Indeed, the blockade of the peripheral IL-6 receptor was shown to impart a rapid and sustained antidepressant effect in a mouse model of depression generated by social defeat. This effect was attributed to the normalization of the GM [76]. Moreover, pre-treatment with pre- or pro-biotics or even fecal microbiota transplantation have been advocated in MDD therapy [77]. Additionally, it has been proposed that examination of the gut microbiota may predict both MDD and cognitive dysfunction, suggesting that the readout of the microbiome may present a noninvasive prognostic tool for MDD and cognitive functions [78].

## 3. Drug Addiction

Drug addiction, defined as compulsive drug seeking despite adverse consequences, is a brain disorder of universal medical and social concern. With approximately 300 million people using illicit drugs, and nearly 40 million people affected by SUD, resulting in over 600,000 deaths annually worldwide, the challenge for public health and the economic burden is staggering [79]. This challenge is exacerbated by the fact that there is a high relapse rate of 50 to 70% despite the advances in our understanding of the neurobiological substrates and availability of newer treatment modalities [80]. Thus, there is an urgent need for more effective therapies.

Drug addiction involves functional changes to brain circuits controlling reward or pleasure and extends to pathways dealing with stress response and self-control [81]. In particular, the mesocorticolimbic DAergic pathway, originating in the ventral tegmental area (VTA) and projecting to the nucleus accumbens (NACC), central amygdala, and medial prefrontal cortex (mFCX), has been referred to as the “reward pathway” due to its intimate association with natural rewards as well as euphoria induced by substances of abuse. Modifications in this circuitry has also been linked to reward dysfunction, drug tolerance, escalation of drug intake, and eventual compulsive use of the drug [82,83,84,85,86].

Although a very strong influence of genetics, where genes may account for about half of a person’s risk of addiction, is evident, experience with drugs, especially at younger or adolescent age, stems from several factors. For example, the use of stimulants such as cocaine is attributed to the initial feeling of euphoria, followed by power, self-confidence, and increased energy, whereas the euphoria caused by opioids is usually followed by feelings of relaxation and satisfaction. In other instances, an addictive substance (e.g., alcohol or nicotine via smoking) may be used to mitigate stress, anxiety, or depression. In addition, curiosity and peer-pressure may be key reasons for trying drugs during the developmental period [87,88,89,90]. In all, negative affects, such as “Hyperkatifeia”, may be a major player in continuing drug use as well as in relapse [91]. In this context, it should be noted that because of the development of tolerance to the drug, more and more would be needed to produce the same effect [82,92].

### 3.1. Neurotrophic Factors and Drug Addiction

The widespread consequences of abusing drugs, resulting in long-lasting behavioral effects, are attributed to the structural and functional changes in both neurons and glial cells. In this regard, BDNF, via regulating phosphatidylinositol 3′-kinase (PI3K), mitogen-activated protein kinase (MAPK), phospholipase Cγ (PLCγ), and nuclear factor kappa B (NFκB) signaling pathways, plays a key role. Indeed, BDNF, via alteration of the reward circuitry, can modulate the motivation to take drugs [82,93]. Interestingly, administration of both BDNF and glial cell line-derived neurotrophic factor (GDNF), discussed in more detail below, increase cravings when administered into the mesolimbic reward pathway [83].

DAergic neurons contain both BDNF and GDNF receptors; however, their therapeutic use is still unclear [83]. It is noteworthy that age, sex, and even age of first drug use influence BDNF levels in patients suffering from SUD. The lower level of serum BDNF levels in SUD subjects has prompted the suggestion to use BDNF as a marker in SUD [94,95].

### 3.2. Neuroinflammation and Drug Addiction

Physiological homeostasis of the body, including that of the brain, is critically dependent on the proper functioning of the immune system. Thus, hyperactivation of the immune system can induce peripheral inflammatory responses as well as central neuroinflammation. The latter leads to dysregulated neuronal processes, which can contribute significantly to SUD [4,96]. In this regard, substances of abuse trigger the release of cytokines such as interleukins (ILs), tumor necrosis factors (TNF), interferons (IFNs), chemokines, and lymphokines from both the immune cells like macrophages, and lymphocytes (e.g., T-cells, B-cells, and natural killer cells) or non-immune cells such as fibroblasts and endothelial cells [4]. The cytokines in turn stimulate their specific receptors and enhance the immune response, leading to neuroinflammation. Some cytokines (e.g., IL-10) may impart anti-inflammatory effects [97].

In the brain, neuroinflammation is underscored by gliosis and microglial activation, which also cause the release of pro-inflammatory factors and, in this case, can compromise the integrity of the BBB and lead to further complications [4,98]. Thus, when neuroinflammation ensues, it may further exacerbate SUD or induce MDD [4,98]. For example, it has been shown that psychostimulants or endocannabinoids activate microglia, causing the release of different pro-inflammatory cytokines and leading to neuroinflammation, which can further contribute to drug abuse [96]. Similarly, mu-opioid receptors (MORs) that are well-known for their role in analgesia and drug addiction have been related to neuroinflammation, where together with toll-like receptors (TLRs), they enhance the release of pro-inflammatory cytokines [99].

On the other hand, some addictive substances such as alcohol may suppress the immune system in some individuals, rendering them susceptible to infection and other diseases [100,101]. Taken together, it is suggested that neuroinflammation promotes addiction-related brain and behavioral deficits and that strategies to reduce neuroinflammation may be viable novel interventions in SUD [4,102].

### 3.3. Gut Microbiota and Drug Addiction

SUD has a reciprocal relation with the GBA and is associated with dysbiosis [103,104,105]. GBA communication is manifested through different mechanisms, including the immune response, where the bacterial products may alter the intestinal barrier allowing such products to enter the bloodstream and induce an inflammatory response. Other bacterial products such as short chain fatty acids (SCFAs) may interact directly with the brain. Finally, some bacterial products may affect the hypothalamic–pituitary–adrenal (HPA) axis, which in turn can activate microglia [103,105]. Among substances of abuse, alcohol causes dysbiosis and peripheral inflammation, and its withdrawal has been shown to cause neuroinflammation [103]. Similarly, chronic morphine or cocaine use result in dysbiosis, increased intestinal permeability, and neuroinflammation, which can, in turn, influence the drug response [103,104].

The brain’s modulation of the gut microbiota occurs primarily through the autonomic nervous system and several neurotransmitters that act directly on bacterial gene expression. In sum, substances of abuse, by causing dysbiosis and oxidative stress, part of which may be due to the alteration of antioxidant capacity, induce neuroinflammation, which can lead to neuropsychiatric/neurodegenerative diseases [105]. For this reason, it has been proposed that the use of “psychobiotics” may be a novel approach in dealing with these devastating diseases [105].

## 4. Glial Cells

Glial cells were first identified in the mid-19th century by the leading neuroscientists of the time, including Santiago Ramón y Cajal and Pío del Río-Hortega. The term neuro-glia (neuro-glue) was coined by the pathologist Rudolf Virchow to signify their role in maintaining the integrity of the neurons. Camillo Golgi described astrocytes and oligodendrocytes in his book in 1871. The term astrocyte was introduced by Michael von Lenhossek in 1893 to reflect their star shape [106,107]. Until relatively recently, glial cells were considered “passive” cell populations that merely provide structural support and sustain neuronal cells. However, glial cells are now considered one of the most versatile cells in the body due to their varied functions, including axonal guidance, proliferation, trophic effects, and maintaining neural function and development. Indeed, glial cells outnumber neurons 10 to 1, representing the biggest cellular population in the brain [108,109,110]. These cells play a critical role not only as energetic support for neurons [66,111,112], but also in the control of metabolism [113,114], myelination [115,116], formation of the BBB [117,118], development and remodeling of synapses [119,120,121], regulation of neurotransmitters [122,123,124], control of fluid/electrolyte homeostasis [125], neuroendocrine function [126], innate immunity response [127,128], and detoxification [129,130]. It is not surprising, therefore, that their disruption or dysregulation can lead to neuropsychiatric and/or neurodegenerative diseases [60,116,131]. Indeed, recent developments suggest potential novel interventions in neurodegenerative diseases by targeting glial receptors and enzymes [132]. We have recently proposed that glial nAChRs may be a suitable target for intervention in Parkinson’s disease (PD) [133]. It would be of interest to determine if this hypothesis can extend to MDD and SUD.

To date, four main glial cell subtypes, including microglia, astrocytes, oligodendrocytes, and synantocytes or NG2 cells, have been characterized. Here, following a brief description of each, we specifically concentrate on their potential roles in MDD and SUD, as glial cells have been implicated in both these disorders [134,135].

### 4.1. Microglia

Although microglia are an important component in the brain’s glia, unlike all other glial cells, they do not originate from the ectodermal tissue but from yolk sac progenitors that are abundant during brain development. Microglia cover a large volume of the parenchyma in adults, amounting to 10–15% of all CNS cells. They constantly survey the environment through rapid movements of their fine filopodia, allowing them to react quickly to any kind of insult. Lately, it has been shown that the action of filopodia is cAMP-dependent [136]. Microglia are considered the immunocompetent and phagocytic cells of the nervous system and share the same origin and express many common cellular markers with peripheral macrophages. Because of their function in innate immune response, they play a key role in neuroinflammation, which, as alluded to above, may be responsible for the manifestation of MDD and SUD. Microglia play a vital role in maintaining brain homeostasis by eliminating cell residues as well as pathogens. For this reason, they are referred to as resident macrophages in the CNS [109,137]. Microglia also regulate the neurogenesis, formation, and elimination of synapses, control the number of neuronal precursor cells, and mediate the infiltration of T-cells into the brain [138].

Initially, two distinct profiles were ascribed to microglia: M1, or a pro-inflammatory state where production of chemokines, cytokines, and metabolites lead to neuroinflammation; and M2, an opposite anti-inflammatory state involved in damage repair and neuroprotection [138,139,140]. However, recent studies suggest that differences in microglia functions are not driven exclusively by their milieu, but, rather, by the unique properties they possess. Therefore, it is suggested that microglial subtype categorization be based on their function [132,141,142].

The activity of microglia consists of three states termed resting, activated, and phagocytic stages. At the resting state, they are highly ramified but not active. Once activated, due to insult or injury, they contract and the cell body enlarges, allowing them to proliferate and assume a full-blown phagocytic characteristic. This serves the purpose of eliminating debris, which is essential for repair and recovery. Overactivation of microglia, however, leads to neuroinflammatory and neurological conditions, including MDD [143,144,145,146].

Microglial polarization into various stages occurs due to perturbation in the microglial micro-environment. In the resting state or under physiological conditions, microglia have a small cell body and very fine and highly ramified processes, which allows them to survey the local environment for cellular damage. This stage is now referred to as a “surveilling” stage rather than resting state [147,148,149]. Upon activation, the cell body enlarges, and microglia processes assume a shorter or amoebic shape, allowing them to quickly reach the site of injury and initiate phagocytic activity. Amoeboid microglia have completely retracted processes with swollen cell soma to facilitate phagocytosis. These morphological transformations may specify disease-specific stages. In mice, a fourth morphology of microglia, referred to as rod-like microglial cells, that exhibit fewer secondary branches and narrowing of the cell soma, have been observed [149,150,151]. Microglia express various receptors including low-density lipoprotein receptor-related protein 1 (LRP1), triggering receptor expressed on myeloid cells-2 (TREM2), calcium-sensing receptor (CASR), nicotinic cholinergic receptors (nAChRs), and toll-like receptors 2 and 4 (TLR2 and TLR4) [133]. TLRs, expressed by both microglia and astrocytes (discussed below), are a well-characterized family of pattern recognition receptors (PRRs) that sense endogenous debris or pathogens and initiate the innate immune response. TLRs contribute significantly to CNS pathology and are under intense investigation for potential therapeutic targets [138,152,153,154,155].

#### 4.1.1. Microglia–Depression

Glial cells in general, and microglia in particular, exert a critical role in MDD. Indeed, this involvement is so significant that it was proposed to designate depression as a microglial disease [156,157], although this terminology might not be universally applicable. The reason for such designation was due to the initial findings showing that microglia regulate synaptic plasticity, the formation of neural networks, and neuroinflammation, all of which play important roles in the manifestation of MDD [156,157]. In the last three decades, it has become evident that patients with chronic inflammation manifest increased levels of circulating cytokines, microglia overactivation, and depressive symptoms. Moreover, microglia, as well as astrocytes and OLs, are responsible for the transfer of exosomes or secreted extracellular vesicles (EVs) to neurons [158]. EVs are key players in intercellular signaling as they carry mRNAs, microRNAs (miRNAs), and specific proteins to the neuron. Interestingly, depressed patients manifest changes in miRNAs, which are known to affect neurotrophic factors, immune cells, synaptic plasticity, cognition, and mood [158]. More recently, using transcriptional profiling, it was revealed that MDD was associated with microglia inhibition in the cortical gray matter [159].

It is also a well-established fact that chronic stress may lead to depression. Stress does this by disrupting homeostasis via its effects on microglia [160,161]. Homeostasis imbalance may include GBA dysregulation and unbalanced pro- vs anti-inflammatory cytokines and neurotransmitters [160].

It is noteworthy that GM maturation, which is critical for neuronal maturation, appears to parallel the temporal course of brain development [162,163]. Although further elucidation of microglia’s role in the etiology of depression is warranted, it can be summed up that microglial dysfunction can lead to a variety of neuropsychiatric diseases including MDD. Indeed, the term “microgliopathy” has been coined to refer to such diseases. Accordingly, a growing number of studies are suggesting that targeting microglia (inhibition or stimulation depending on the microglial status) could be a novel personalized medical approach in these devastating diseases [156,164].

#### 4.1.2. Microglia–Drug Addiction

The critical function of microglia and astroglia in synaptic formation and refinement is well-recognized. Drugs of abuse, on the other hand, cause persistence alterations in synaptic and neuronal function. Thus, growing evidence suggests that disruptions in glial function may be associated with SUD [165,166]. Specifically, glial and neuroimmune mechanisms are believed to contribute to opioid, alcohol, and psychostimulant abuse. It is postulated that microglia and astrocyte activation by drugs of abuse occur via stimulation of innate immune receptors, which result in the secretion of chemokines and cytokines, which in turn influence neuronal function [165,166]. The stimulation of innate immune receptors may also lead directly to synaptic remodeling [165,166].

More recently, a xenobiotic hypothesis has been advanced which posits that substances of abuse are exogenous molecules and are therefore considered foreign “invaders” that trigger the protective immune response. Microglia, as the primary resident immune cells in the brain, provide the initial defense mechanism. However, with persistent and repeat administration of substances of abuse, the overactivation of microglia and a neuroinflammatory condition ensues that, in turn, can further contribute to drug addiction via modulation of neuronal function [167]. A recent report indicating the necessity of microglia in NACC synaptic adaptations during cocaine withdrawal further supports the importance of these cells in drug addiction [168].

Taken together, it is evident that further inquiries into glial–neural interactions can not only enhance our understanding of SUDs but may also provide novel therapies.

### 4.2. Astroglia (Astrocytes)

The term astrocyte or astroglia was coined by the Hungarian anatomist and histologist Michael von Lenhossék in 1895 due to the star-like shape of these cells [169]. Since then, based on morphology and spatiotemporal distribution and function, various subpopulations have been identified. Depending on the brain area, astrocytes may constitute anywhere between 17 and 61% of the total cells. Like microglia, they exhibit heterogeneous phenotypes in response to various insults, commonly referred to as astrocyte reactivity [153]. Astrocytes play a crucial role in maintaining neuronal integrity and function, as they form synapses with neurons and, at least for glutamatergic neurons, are critical in the reuptake of this excitatory neurotransmitter [169,170]. In addition to providing structural integrity to the extracellular matrix, astrocytes monitor and regulate pH homeostasis, provide nutrients, remove waste, and are a key component and regulator of the BBB [169,170].

Interestingly, glial cells contain their own neurotrophic factor, referred to as glial cell line-derived neurotrophic factor (GDNF), a class of proteins that also provide trophic support to neuronal cells, including DAergic neurons [171]. Indeed, it has been proposed that lacrimal GDNF may serve as a marker in MDD [172]. Astrocytes also express a high level of glial fibrillary astrocytic protein (GFAP), which are commonly used as a marker for their identification, although lately other markers have been added to the list [173]. More recently, it was reported that astrocytes are the necessary source of TNF-α for the mediation of homeostatic synaptic plasticity [174]. Similarly, taurine, considered the most abundant free amino acid in the brain required for optimal postnatal brain development, occurs predominantly in astrocytes. Taurine has antioxidative and anti-inflammatory functions, hence, cytoprotective properties [175].

Unlike microglia, astrocytes do not contain receptors that recognize pathogens; however, they do become reactive when activated by polarized microglia, and they release inflammatory mediators and modulate inflammation [138].

Thus, it may be suggested that astrocytes work together with microglia to provide the first line of defense against insults. However, overstimulation of proinflammatory signals may synergistically contribute to neuronal dysregulation and ensuing neuropsychiatric/neurodegenerative diseases [176,177,178].

In addition to the intimate interaction between astrocytes and microglia, an interaction between astrocytes and neurons, referred to as crosstalk, has been identified [153,171,179,180]. It is anticipated that as our knowledge of such crosstalks expands, novel interventions in neuropsychiatric/neurodegenerative diseases including MDD and possibly SUD may be realized [153,180,181].

#### 4.2.1. Astrocytes–Depression

Numerous studies, including ones on the analysis of postmortem brain tissue, have confirmed a central role for astrocytes in MDD pathology. Thus, human postmortem studies demonstrate significant reduction in GFAP in the gray matters of the dorsolateral PFCX and the orbitofrontal cortex, the white matters of the anterior cingulate cortex, the orbitofrontal cortex, and the CA1 and CA2 subregions of the hippocampus of depressed individuals [182]. It is of relevance to note that GFAP, which is used as a primary marker of astrocytes, helps maintain the shape, mechanical strength, cell movement, and astrocyte–neuron communication of astrocytes [182]. In animal models of stress-induced depression, significant reductions in cortical and hippocampal astrocytes were also noted. Interestingly, the effects of chronic stress on astrocytes could be reversed by antidepressant treatment. Specifically, astrocytes’ intracellular signaling pathways, receptor expressions, release of various trophic factors, and gene expression were restored by antidepressants, suggesting that the efficacy of the treatment with antidepressants also involves astrocyte modifications [183].

The reduction in astrocytes is a key feature in MDD pathology and has also been confirmed in other animal models of depression. It is argued that astrocyte dysfunction through the disruption of astrocyte–neuron interactions affects the neurotrophic function, monoaminergic transmission, and the excitatory–inhibitory balance of local networks, leading to mood disorders [184,185]. Interestingly, the reduction in the number of astrocytes in MDD has been partially attributed to potassium imbalance, and animal studies suggest that the antidepressant effects of ketamine might also be due to the correction of this imbalance [186]. The reduced astrocyte densities in MDD were correlated with elevated S100 beta (S100B) in cerebrospinal fluid levels. S100B is a multifunctional protein that is expressed in large amounts in protoplasmic astrocytes [187] and in myelinating oligodendrocytes [188]. It is a Ca^2+^-binding protein necessary for neurotrophic functions and may therefore be involved in neurodegenerative diseases and/or MDD [186].

It is of relevance to note that astrocytes also express monoaminergic transporters that are involved in modulating neuronal activity [189]. Moreover, it has been suggested that the antidepressant effects of SSRIs may be largely due to activation of BDNF mRNA and an increase in BDNF levels in astrocytes [189,190]. Curiously, a recent study using an experimental model of periodontitis-induced depression in mice revealed that SorCS2, a gene that controls the expression of a critical amino acid transporter [191], is responsible for the expression of depressive phenotype in these mice [192]. Interestingly, SorCS2 was shown to also be involved in pro-BDNF and glutamate signaling in the hippocampus [192].

Recent studies indicate that the dysregulation of the astrocytic purinergic system contributes to the pathophysiology of MDD. Thus, the overactivation of adenosine A2A receptors (Rs), P2X7, P2Y1, and P2Y11Rs results in neuroinflammation, disruption of neuro–glia communication, and synaptic plasticity in depression-relevant areas of the brain such as the hippocampus, medial prefrontal cortex, and amygdala. Curiously, astrocytic A1Rs may impart neuroprotective and immunosuppressive effects, which could present novel intervention targets in MDD [193,194].

More recently, the revelation of the importance of astrocyte Ca^2+^ signaling in the control of homeostasis in brain circuits and behavioral regulation has led to the suggestion that we can potentially exploit this system in the treatment of MDD [195,196]. Moreover, it is now well documented that neurons and astrocytes through gliotransmission may contribute to depression by influencing synaptic plasticity as well as energy metabolism [195,196,197]. Thus, further understanding of astrocyte-neuron involvement in MDD pathology, including alterations of pyramidal neuron and mid-spiny neurons, as well as alterations of gliotransmitters between astrocytes and neurons, could provide novel targets for MDD [195,197,198,199].

#### 4.2.2. Astrocytes–Drug Addiction

The role of astrocytes in synaptic functions and glutamate clearance are well established. It is also known that substances of abuse compromise this capacity and facilitate relapse. For example, astrocytes in the NACC undergo rapid and transient plasticity in response to drug-associated cues [200]. Several key astrocytic signaling pathways that are involved in cocaine-induced synaptic and circuit adaptations have been identified [201,202]. Overall, it is postulated that NACC astrocytes play a critical role in modulating glutamate transmission during relapse [199,200,201]. Moreover, the therapeutic potential of targeting astrocytic substrates to tackle drug addiction has been proposed [132,203,204].

More recently, observations of changes in gene expression, mediated by transcriptional and epigenetic regulations brought about by drugs, have provided fresh venues for potential therapeutic interventions. In this regard, evidence of the robust transcriptional response of astrocytes to several substances of abuse, whereby astrocytes are directly implicated in drug seeking behaviors, has been provided [203,204,205].

### 4.3. Oligodendrocytes

Another major glial cell consists of oligodendrocytes (OLs), which are now well recognized as the primary source of myelination in the CNS [206]. In the adult CNS, 75% of all glial cells are OLs. OLs are not only intimately involved in axonal myelination, but they also regulate extracellular concentration of potassium, provide trophic (e.g., GDNF and BDNF) and metabolic supply for myelin, and modulate the growth of axons [206,207]. These functions underscore their importance in CNS functioning. OLs, like micro- and astroglia, express TLRs, which are important in the formation of myelin [115,208,209]. Importantly, both MDD and SUD are associated with glial cells dysregulation. Hence, we delve into these connections below.

#### 4.3.1. Oligodendrocytes–Depression

As far back as two decades ago, the involvement of OLs in depression was verified by the finding that glial reduction in the amygdala in MDD was due to oligodendrocytes loss [210]. Later, reductions in the density and ultrastructure of OLs were also detected in the PFCX and amygdala of MDD patients [211]. It was hypothesized that pathological changes in OLs were due to the disruption of white matter tracts, and that factors such as stress, altered gene expression of neurotrophic factors, and glial transporters modify glial cell number and affect the neurophysiology of depression [212]. Further evidence was provided through the demonstration of how genetic alterations in these cells alone can result in major behavioral changes including MDD [213].

Recently, some clues as to the cellular and molecular bases of white matter pathology have been revealed. Thus, it has been shown that oxidative damage was a major culprit in MDD and that interference with this pathway could be of potential benefit [213]. Furthermore, in a repeat defeat mouse model of depression, long-lasting OPC losses, aberrant differentiation into OLs, and severe hypomyelination were observed in the PFCX [214]. In addition, morphological impairments in OPCs, excessive oxidative stress, and OL apoptosis were noted in these mice [214]. Markers of oxidative stress were also observed in OLs obtained from the brainstem and occipital cortex of MDD patients [215]. Single-nucleus transcriptomic data analysis revealed OL dysregulation and the presence of novel OLs expressing immune properties in MDD patients [216]. It is expected that further elucidation of the interaction between OLs and neurons will provide novel insights into the pathology of MDD and suggest novel targets for intervention [217].

#### 4.3.2. Oligodendrocytes–Drug Addiction

Although the effects of drugs on myelination and white matter integrity have been reported, specific effects on OLs are yet to be investigated. Nonetheless, by applying a transcriptome analysis, it was shown that heroin self-administration in rats upregulates markers of OL maturation and differentiation [218,219]. Moreover, opioids may directly or indirectly regulate OL development and myelin generation in the PFCX of rats during prenatal, adolescence, and young adulthood [219]. Interestingly, detrimental effects of prenatal alcohol exposure on OPC and OL lineage were recently detected in human fetal autopsies [220].

### 4.4. Synantocytes (NG2 Cells)

Synantocytes, also referred to as neuron glial 2 or nerve/glial antigen 2 (NG2) cells, are the fourth subset of major glial cells in CNS. These cells are OL-precursor cells and are usually identified by the presence of two key markers: platelet-derived growth factor receptor alpha (PDGFRα), and the chondroitin sulfate proteoglycan NG2 [204,221]. NG2 cells are almost uniformly distributed in both white and gray matter areas, exhibit a complex stellate morphology, tend to associate closely with neuronal dendrites and cell bodies, and keep their ability to maintain proliferation in the adult brain [204,221,222].

While NG2 cells were initially thought to be solely OL progenitors, later, their capacity to give rise to astrocytes and neurons were verified [204,221,222]. Additionally, their potential influence in a variety of conditions, including neurodegenerative diseases, such as AD, multiple sclerosis, traumatic brain injury, epilepsy, glioma, and mood disorders, were investigated [223,224]. NG2 cells were also implicated in experimental autoimmune encephalomyelitis (EAE), a disease associated with increased BBB permeability and neuroinflammation [225]. The mechanism of their action was postulated to be via reactive T-cell stimulation and the controlling of IL-12 expression [225]. NG2 cells may also play a critical role in the modulation of neuroinflammation [226] and neurovascular unit formation during development [227]. Their importance in the generation of OLs and angiogenesis following acute ischemic stroke was also recently reported [227]. Importantly, their communication with neurons and their influence on neuronal plasticity and various behaviors makes them a potential target for therapeutic interventions in a variety of diseases [228,229,230].

#### 4.4.1. NG2 Cells–Depression

NG2-glia are heterogeneous glial cells with distinct roles in neuronal plasticity, the dysfunction of which can lead to neurological and behavioral abnormalities [229]. Indeed, the influence of NG2 cells in stress-induced mental disorders, including depressive-like behavior, was recently highlighted [230]. It was concluded that further inquiry into the causal role of NG2 cells in stress response and stress-related psychopathologies would be required before novel interventions in such behavioral disorders can be recommended [231]. NG2 cells, however, have been proposed as a potential target in the prevention of epilepsy following viral infection [231,232]. Moreover, it was revealed very recently that NG2 cells, in the cortex of adult mice, may act as progenitors to neural cells [232]. If verified in human neuronal system, the substantive exploitation of NG2 cells in neuropsychiatric and/or neurodegenerative diseases may become feasible [232].

NG2 cells exert substantial influence in the modulation of neuroinflammation [225]. This, together with the observation that the ablation of NG2 cells exacerbates dopaminergic neuronal cell loss, suggest that NG2 cells may act as negative regulators of neuroinflammation [226]. Given the causal relationship between neuroinflammation and MDD, a role of NG2 cells in this disorder is anticipated. Interestingly, it was recently reported that exosomes derived from dental pulp stem cells may promote NG2 cells proliferation and differentiation [227]. Exosomes are small, extracellular vesicles secreted by various stem cells and are potent mediators of intercellular communication and tissue repair [233,234]. Indeed, clinical applications of exosomes in general surgery, neurosurgery, orthopedic surgery, head and neck surgery, cardiothoracic surgery, plastic surgery, acute skin wound healing, urology, ophthalmology, obstetrics and gynecology, and other diseases induced by cancer, ischemia, or inflammation have been suggested [235,236]. Whether their application in MDD may also be viable awaits further investigation.

#### 4.4.2. NG2 Cells–Drug Addiction

Although very limited literature on the direct involvement of NG2 cells in SUD is available, almost a decade ago, their potential influence in drug addiction was reviewed [237]. It was argued that further investigation of gliogenesis or new glia generation by NG2 cells in the cortex would be of relevance to SUD, as NG2 cells may have a role in negative reinforcement and relapse [237].

More recently, studies in human fetal brain have confirmed animal findings relevant to fetal alcohol syndrome (FAS), where dysregulated cytokine expression, apoptosis of NG2 cells, and altered OL differentiation were observed following prenatal alcohol exposure [238]. Thus, the interactions of drugs of abuse with NG2 cells are verified. It is anticipated that further investigation of the mechanisms involved in these interactions would yield novel therapeutic targets [218].

## 5. Glia–Neuron Interaction–SUD

It is noteworthy that practically all drugs of abuse affect glial–neuronal interactions. For example, psychostimulants such as cocaine and methamphetamine, or opioids such as morphine, have been shown to increase glial cell reactivity and increase the levels of proinflammatory cytokines such as Il-1β, TNFα, and IL-6, but decrease the levels of anti-inflammatory cytokines such as IL-10 [138,239,240,241]. Moreover, substances of abuse may reduce astrocyte cell number, alter glutamate neurotransmission and metabolism through the specific astrocytic glutamate transporter GLT-1 [242,243], increase the number of reactive microglia and neuroinflammatory markers, as well as impair OLs, resulting in a decrease in myelination. Finally, glial dysfunction may affect BBB permeability and contribute to neurotoxicity, further exacerbating addictive behavior [138].

Drugs of abuse affect glial cell activities, resulting in perturbations in specific brain regions/circuitries associated with SUD. These include PFCX, as well as the mesolimbic reward pathway. In this regard, the crucial role of astrocytes in the reuptake of glutamate, which plays an important role in the development and expression of addictive behaviors, is well recognized [244,245,246,247]. Thus, astrocytes can regulate the activity of neurons by releasing chemicals and gliotransmitters as well as controlling extracellular concentrations of ions and neurotransmitters [246,247]. Together, these results indicate that substances of abuse can cause neuronal injuries in the reward system via their action on glial cells [201,248,249].

### Therapeutic Interventions

SUD may lead to lasting neuroadaptations in corticostriatal projections, a trajectory believed to be critical in inhibitory control over addictive behavior. Indeed, it is suggested that disruptions in glutamate homeostasis following chronic drug use is a major factor in relapse [201,248,249]. Beta-lactam antibiotics, including cefazolin, amoxicillin, ampicillin, cefoperazone, and ceftriaxone, which has been best studied, stimulate GLT1 expression and function [250]. Thus, it has been shown that restoration of GLT1 by ceftriaxone inhibits cue- and cocaine-induced reinstatement and suppresses alcohol dependence [251,252,253]. More recently, it was reported that a novel GLT1 modulator has similar effects in a rat model of alcohol drinking [254].

In addition to GLT1 modulation, neurotrophic manipulation of glial cells may also provide a novel intervention in SUD. In this regard, GDNF could be a viable target for SUD treatment [255]. However, poor BBB permeability of GDNF limits its clinical applicability [160]. Moreover, it has been shown that BDNF applied to PFCX reduces cocaine seeking [255]. Curiously, this effect was attributed in part to normalization of glutamate homeostasis in the NACC [256]. An interaction between GLT1 and neurotrophic factors is further supported by the results of a recent study showing that GLT1 modulation may directly affect neuroinflammation and neurotrophic biomarkers in the mesocorticolimbic reward pathway of alcohol-preferring rats [254].

**Figure 1:**
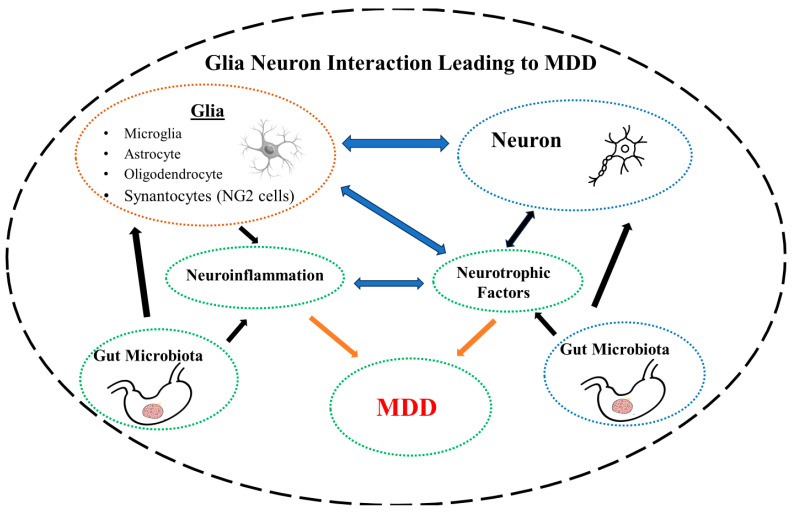
Schematic diagram depicting glial–neuron interaction, disruption of which by defects in neurotrophic factors or dysbiosis of the gut microbiota may lead to neuroinflammation and major depressive disorder (MDD).

**Figure 2:**
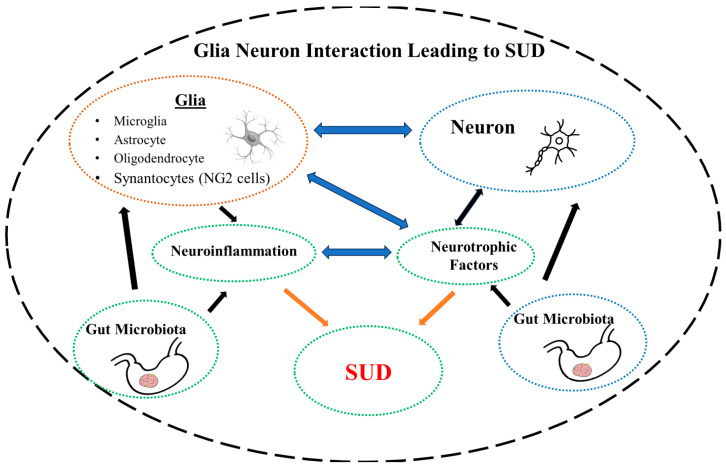
Schematic diagram depicting glial–neuron interaction, disruption of which by defects in neurotrophic factors or dysbiosis of the gut microbiota may lead to neuroinflammation and substance use disorder (SUD).

In summary, glial–neuron interaction is influenced by NTFs as well as GM. Disruption of this intricate balance due to changes in NTFs or GM may lead to neuroinflammation and MDD (Figure 1) or SUD (Figure 2).

## 6. Microbiota–Neurotrophic Factors–Neuroinflammation

It is noteworthy that there exists an intimate interaction between the major factors that affect MDD and SUD. This interaction, while adding to the complexity of the overall picture, may also suggest novel interventions. For instance, butyrate, a SCFA metabolite of the GM, is critical in maintaining proper gastrointestinal and GBA integrity. Disruption of its function can lead to MDD [257,258]. Conversely, sodium butyrate can exert antidepressant effects. This property is attributed to its ability to both inhibit HDAC [217,259,260,261] and increase central levels of BDNF [262]. The antidepressant effectiveness of butyrate was also shown in two animal models of depression, one induced by maternal deprivation and the other via chronic mild stress. Interestingly, in both cases, butyrate increased the central levels of both BDNF and GDNF [261,262,263].

Neurotrophic factors, as alluded to earlier, are essential in maintaining homeostasis of the CNS as they regulate the differentiation, maturation, development, and survival of neurons. They also may control glial cell functions by directing them toward an anti-inflammatory and neuroprotective phenotype [264]. Cytokines, which are key mediators of the immune response, are also involved in communications between the immune system and the CNS. It is postulated that damage produced by over-expression of cytokines dysregulates neuroplasticity by disrupting BDNF and leads to a variety of neurological and/or neuropsychiatric conditions including MDD [265]. This hypothesis was verified recently by the findings that neuroinflammation leads to dysregulated neurogenesis, particularly in the DG of the hippocampus [266].

It is anticipated that as our knowledge of the relationship between GM, GBA, and the role of SCFAs in mood disorders expands, more therapeutic interventions by manipulating GM will become available [267,268,269].

## 7. Concluding Remarks

MDD and SUD are complex brain disorders involving intricate neuronal circuitries that may be influenced by a plethora of factors including genetics, environment (either directly or indirectly via epigenetics), immune system, metabolic products, neurotrophins, gut microbiota, non-neuronal glial cells, etc. The complexity gets compounded by the complicated interactions between the factors.

In this review, we have highlighted the influence of some of the major players such as neurotrophic factors, neuroinflammation (immune system dysregulation), dysbiosis, and glial cells (all four major subtypes) in these devastating disorders. Although we covered in some detail the contribution of glial cells and their primary influence on neuroinflammation, interactions of these cells with other major players such as neurotrophins or gut microbiota cannot be ignored. The grand jigsaw puzzle is perhaps beyond comprehension now. However, placing even a few pieces in their right place helps towards completing the final picture.
